# Hydrogen Bonds, Topologies, Energy Frameworks and Solubilities of Five Sorafenib Salts

**DOI:** 10.3390/ijms22136682

**Published:** 2021-06-22

**Authors:** Chiuyen Phan, Jie Shen, Kaxi Yu, Jiyong Liu, Guping Tang

**Affiliations:** 1 Faculty of Chemical Technology–Environment, The University of Danang—University of Technology and Education, Danang 550000, Vietnam; 2 Department of Chemistry, Zhejiang University, Hangzhou 310028, China; shenjie1003@zju.edu.cn (J.S.); yukaxi@zju.edu.cn (K.Y.); liujy@zju.edu.cn (J.L.)

**Keywords:** sorafenib, hydrogen bond, topology, energy framework, solubility

## Abstract

Sorafenib (Sor) is an oral multi-kinase inhibitor, but its water solubility is very low. To improve its solubility, sorafenib hydrochloride hydrate, sorafenib hydrobromide and sorafenib hydrobromide hydrate were prepared in the mixed solvent of the corresponding acid solution, and tetrahydrofuran (THF). The crystal structures of sorafenib hydrochloride trihydrate (Sor·HCl.3H_2_O), 4-(4-{3-[4-chloro-3-(trifluoro-methyl)phenyl]ureido}phenoxy)-2-(*N*-methylcarbamoyl) pyridinium hydrochloride trihydrate, C_21_H_17_ClF_3_N_4_O_3_^+^·Cl^−^.3H_2_O (I), sorafenib hydrochloride monohydrate (Sor·HCl.H_2_O), C_21_H_17_ClF_3_N_4_O_3_^+^·Cl^−^.H_2_O (II), its solvated form (sorafenib hydrochloride monohydrate monotetrahydrofuran (Sor·HCl.H_2_O.THF), C_21_H_17_ClF_3_N_4_O_3_^+^·Cl^−^.H_2_O.C_4_H_8_O (III)), sorafenib hydrobromide (Sor·HBr), 4-(4-{3-[4-chloro-3-(trifluoro-methyl)phenyl]ureido}phenoxy)-2-(N-methylcarbamoyl) pyridinium hydrobromide, C_21_H_17_ClF_3_N_4_O_3_^+^·Br^−^ (IV) and sorafenib hydrobromide monohydrate (Sor·HBr.H_2_O), C_21_H_17_ClF_3_N_4_O_3_^+^·Br^−^.H_2_O (V) were analysed. Their hydrogen bond systems and topologies were investigated. The results showed the distinct roles of water molecules in stabilizing their crystal structures. Moreover, (II) and (V) were isomorphous crystal structures with the same space group P2_1_/n, and similar unit cell dimensions. The predicted morphologies of these forms based on the BFDH model matched well with experimental morphologies. The energy frameworks showed that (I), and (IV) might have better tabletability than (II) and (V). Moreover, the solubility and dissolution rate data exhibited an improvement in the solubility of these salts compared with the free drug.

## 1. Introduction

Sorafenib, an oral drug, is a multi-kinase inhibitor with anti-tumour activity against a large range of cancers [1]. It is approved for the treatment of patients with advanced renal cell carcinoma, advanced hepatocellular carcinoma and thyroid carcinoma [2]. By regulating the growth, proliferation and preventing the formation of neovascularization in tumour tissues, Sor could prohibit the growth of tumour cells [3,4]. Sor is classified as a BCS (Biopharmaceutics Classification System) class II drug, whose aqueous solubility is very low [5,6]. Therefore, strategies to improve the solubility of Sor are of great clinical importance.

There are many methods for improving the aqueous solubility of drugs, and the most widely used method in the pharmacy industry is salt formation [7], which keeps the intrinsic pharmacological properties of drugs undisturbed [8]. Therefore, a lot of salt forms, such as hydrochloride, hydrobromide, sulphate, maleate, fumarate, tosylate, etc., have been investigated and applied to drug therapies. Among them, hydrochloride salt is the most commonly used one in clinical for its low toxicity and high biocompatibility. Sorafenib hydrochloride hydrated salts, sorafenib hydrobromide and its hydrated salts have been prepared and characterized in the literature [9,10]. However, their crystal structures are not reported until now.

Furthermore, the energy framework combines the efficient calculation of accurate intermolecular interaction energies with a graphical representation of their magnitude. This approach is applied to explain the tabletability properties of the drug crystals, which can be addressed in terms of the anisotropy of the topology of pairwise intermolecular interaction energies [11].

In this study, to explore crystal structures of sorafenib hydrochloride hydrate, sorafenib hydrobromide and its hydrate, we prepared their single crystals from the mixed solvent of acid solution and THF. Unfortunately, the sorafenib hydrochloride hydrated form and its solvated form were crystallized simultaneously. Sorafenib hydrobromide hydrate crystals also formed concomitantly with sorafenib hydrobromide. This concomitant occurrence of crystals has been well explained by the subtle interaction between kinetic and thermodynamic factors [12,13]. In this study, to separate, identify these forms, and consequently remove tetrahydrofuran form, we adjusted the ratio of THF: acid solution in mixed solvent and the temperature of crystallization experiments. The theoretical morphologies of these forms based on the BFDH model were also predicted. The role of water molecules in stabilising the crystal lattice was analysed, and the topologies were described for understanding the hydrogen bond systems. Furthermore, the tabletability was predicted based on their energy framework; and their solubilities, dissolution rates were determined and compared with free sorafenib.

## 2. Results

### 2.1. Concomitantly Crystallization

**Figure 1:**
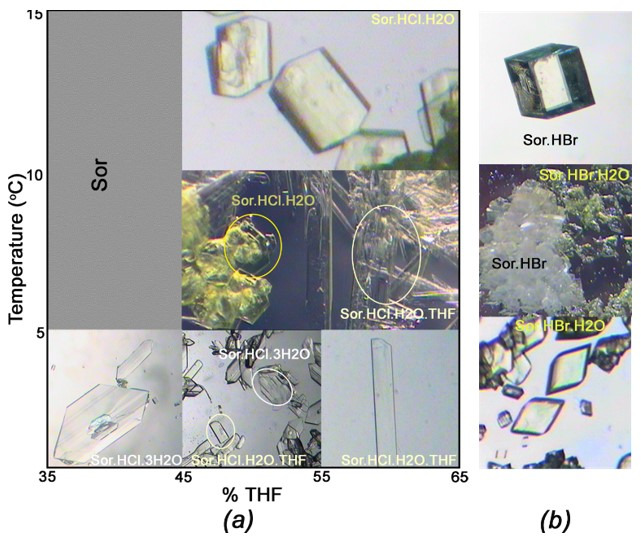
The optical micrographs of (**a**) (I)–(III) crystallized forms in different %THF and temperature conditions, (**b**) (IV) and (V) crystallized forms.

To study sorafenib hydrochloride hydrate (Sor.HCl hydrate), Sor was crystallized from the mixed solvent of hydrochloric acid solution and THF with %THF of 45–55% and temperature at 0–5 °C. Unexpectedly, the concomitant forms exhibiting distinct morphologies and colours were gathered, namely colourless-plate denoted as form (I) and pale yellow-needle as form (III). To eliminate this phenomenon, the altered ratio of THF:water and various temperatures of crystallization were then attempted. The results showed that the obtained crystals significantly changed at different %THF and temperature conditions (Figure 1a). In details, during the preparation of Sor hydrochloride at a low temperature of 0–5 °C, in mixed solvents with %THF of 35–45% and %THF of >55%, as expected, only form (I) or form (III) was formed, respectively. However, in a mixed solvent with %THF 45–65% and temperature at 5–10 °C, concomitant salts of the form (II) and form (III) reappeared, with form (II) being yellow-block. When the temperature was above 10 °C and % THF was higher than 45%, all of the harvested crystals were form (II).

At this point, the purity of three compounds was monitored by the experimental PXRD patterns compared with the PXRD patterns simulated from the crystal structures. As expected, the overlay of three experimental PXRD patterns matched very well with three simulated PXRD patterns (Figure S1), demonstrating that the separation was successful.

With regard to (IV) and (V), when we recrystallized Sor from the mixed solvent of hydrobromic acid solution and THF, only a few crystals of (V) occasionally appeared and crystallized concomitantly with (IV) during the preparation (Figure 1b). Crystal (V) also exhibited yellow-block similar to (II). The structure of crystal (V) was studied by SC-XRD, but its quantity was rather limited, thus further characterizations and properties were hampered.

### 2.2. Morphology

**Figure 2:**
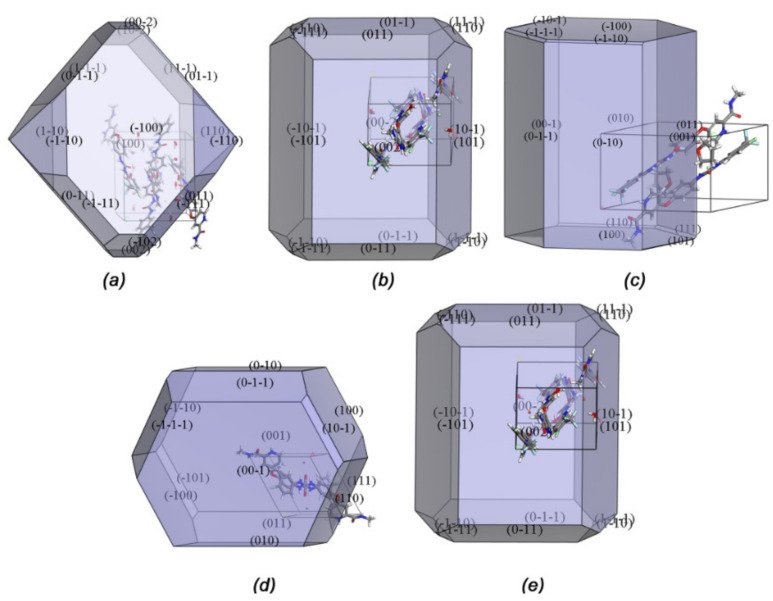
Simulated BFDH morphologies of (**a**–**e**) (I)–(V).

To yield the high-quality crystals, we reduced the presence of concomitant phenomenon in the crystallization process and classified the crystals into three sets based on the morphologies and colours, which could be observed under a microscope. The experimental morphologies of three crystals could be determined compared with the theoretical morphology. Here, we predicted the morphologies of these salts based on the Bravais-Friedel-Donnay-Harker (BFDH) model using Materials Studio (Figure 2). The morphologies of salts (I)–(V) with the Miller indices (MI) superimposed on the face area displayed significant differences. The predicted crystal morphology matched well with the experimentally achieved shape.

The largest faces of most of crystal (I) were (100) and presented as a hexagonal plate. Some of the crystals exhibited hexagonal block, which was identical to the simulated morphology. Whereas, most of crystals (II) and (V) grown from the different THF:water solvents as well as temperatures were multifaceted prisms, with the largest faces being (002) or (00-2). With regard to crystal (III), when it is crystallized in 55–65% at 0–5 °C or 45–65% at 5–10 °C, bulk crystal (III) with needle morphology were obtained, resulting from the fastest growth of (100) facet. In comparison, the crystals were shorter when crystallized from a different condition (%THF of 45–55% at 0–5°), which might be explained by the varied fastest growth faces. The largest faces of most of crystal (IV) was (001), presented as a rhombic block.

### 2.3. Crystallization

In all crystal structures of salts, the proton from the acid (HCl or HBr) transferred to Sor molecule (protonation at pyridine N3 forming pyridinium), resulting in a Sor-H^+^ cation [14].

**Figure 3:**
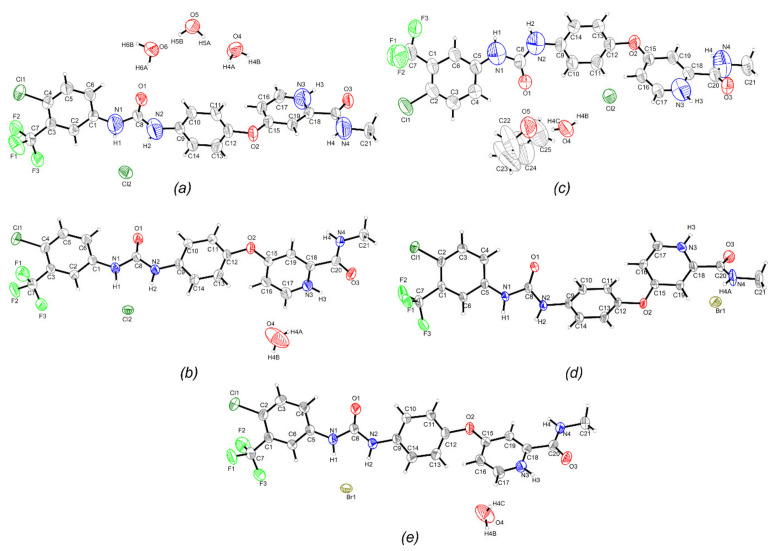
The asymmetric unit of (**a**) (I), (**b**) (II), (**c**) (III), (**d**) (IV) and (**e**) (V) with the atom-labelling scheme. Displacement ellipsoids are drawn at a 50% probability level.

The SC-XRD analysis evidenced that the salt (I) belonged to the P2_1_/c space group (monoclinic) with one protonated drug cation, one chloride anion and three water molecules in the asymmetric unit. While (II) and (V) crystallized in the monoclinic system with space group P2_1_/n. The asymmetric unit of (II) included one protonated drug cation, one chloride anion and one water molecule; and that of (V) had one protonated drug cation, one bromide anion and one water molecule. (III) and (IV), on the other hand, grew in the space group P$\bar{1}$ (triclinic) (Table 1). The asymmetric unit of (III) had one protonated drug cation, one chloride anion, one water molecule and one tetrahydrofuran molecule; and that of (IV) possesses one protonated drug cation and one bromide anion. Figure 3 exhibited the asymmetric unit of (I)–(V), with atom labelling.

### 2.4. Hydrogen Bond

In three hydrochloride salts, the protonated Sor cation had a similar conformation to that in Sor.HCl [14], resulting in a burst of hydrogen bonds. The drug cations were not only linked by the chloride counterions but also water molecules or THF molecules. However, different from Sor.HCl, drug cations in hydrate or solvate forms were not linked to each other by means of the typical hydrogen bond. These hydrogen bonds played a dominant role in administering the crystal structure.

**Figure 4:**
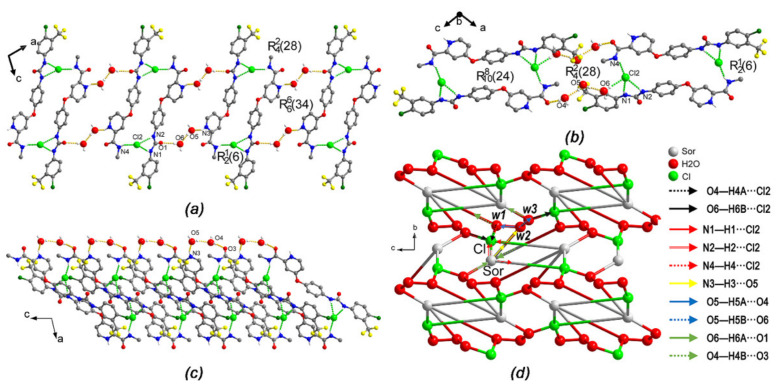
Part of the crystal packing of (I) view along (**a**) [101], (**b**) [10–1] and (**c**) [001] direction, hydrogen bonds are shown as green and yellow dashed lines, H atoms not involved in hydrogen bond have been omitted for clarity. (**d**) 3,3,3,4,5-connected pentanodal topological net representing the hydrogen bond chain structure of (I).

In the crystal structure of (I), the drug cations had four hydrogen bond donor groups (N–H) and two hydrogen bond acceptor groups (C=O) (Figure 4). The N1–H1, N2–H2 and N4–H4 groups were involved in three N–H…Cl hydrogen bonds, creating two ring motifs $R_{2}^{1}\left(6\right)$ and $R_{4}^{2}(28$) [15,16,17]. Consequently, the system created by this ring motif was observed as a centrosymmetric dimer-like structure (Sor.HCl-dimer). Besides that, N3 on pyridinium ring formed N3–H3⋯O5 hydrogen bond with a water molecule (*w2*). The C=O groups were hydrogen bond acceptor in the O6–H6⋯O1 and O4–H4B⋯O3 hydrogen bonds. On the other hand, the chloride anion acted as an acceptor in the hydrogen bond (Table 2). Two anions were located between two drug molecules and two water molecules (*w1* and *w2*), forming five hydrogen bonds for each fragment. Two water molecules were hydrogen bond donors in O–H⋯N and O–H⋯Cl hydrogen bonds, and they were also hydrogen bond acceptors in O5–H5A⋯O4 and O5–H5B⋯O6 hydrogen bonds with the third water molecule (*w3*). These complex hydrogen bond systems resulted in a three-dimensional (3D) network, and it can be readily deciphered into three simple 1D substructures. In the first substructure, the connections of *w2* and *w3* to the Sor.HCl-dimer, creating a 34-members $R_{6}^{6}\left(34\right)$ motif, formed a *zigzag* chain running parallel to the [101] direction in which each Sor-HCl-dimer is a centrosymmetric point (Figure 4a). In the second substructure, the combination of the Sor.HCl-dimer, *w1*, *w2* and *w3* created an out-of-straight infinite chain parallel to the [10–1] direction; meanwhile one drug cation of the Sor.HCl-dimer was connected to *w1*, and one chloride anion of that was linked to *w3* (Figure 4b). These two chains crossed each other resulting in a surface parallel to the {010} plane as a two-dimensional sheet. The third substructure was a V-shaped infinite chain parallel to the [001] direction (Figure 4c). This chain was formed by one Sor.HCl-dimer, one *w1* and one *w2* through O4-H4…O3, O5-H5…O4 and N3-H3…O4 hydrogen bonds. Taken together, the complex hydrogen bond system of form (I) was a pentanodal 3,3,3,4,5-connected net whose topology could be described as F3_3_.3_3_.3_3_.5_4_.6_5_ (Figure 4d).

**Figure 5:**
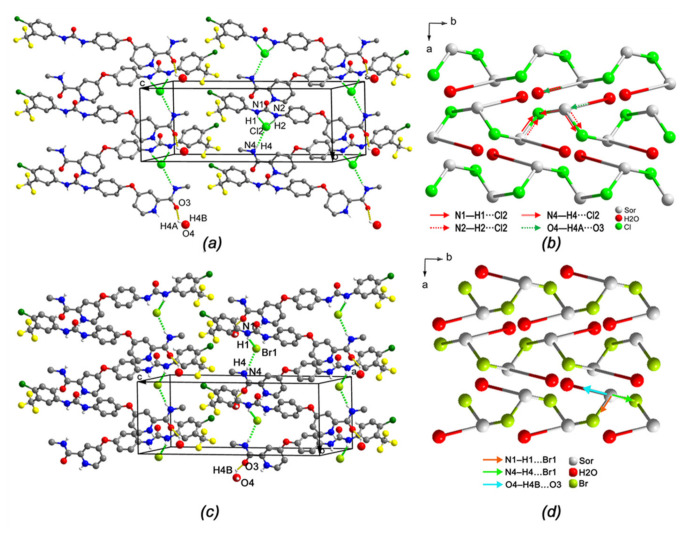
(**a**,**c**) part of the crystal packing of (II) and (V), hydrogen bonds are shown as green and yellow dashed lines, H atoms not involved in hydrogen bond have been omitted for clarity. (**b**,**d**) 1,2,3-Connected trinodal topological 1D net representing the hydrogen bond chain structure of (II) and (V).

With regard to two isomorphous crystals (II) and (V), they crystallized in the same monoclinic system with space group P2_1_/n. Their unit-cell parameters and the cell volumes of two salts are found to be similar. The one-dimensional (1D) chain structure of (II) was formed by three hydrogen bonds with the chloride counterion involved, including N1-H1⋯Cl2, N2-H2⋯Cl2 and N4-H4⋯Cl2 (Table 3). The two former linked result in a six-membered $R_{2}^{1}\left(6\right)$ ring motif (Figure 5a). That of (V) was formed by N1-H1⋯Br1 and N4-H4⋯Br1, due to the long diameter of bromide counterion (Table 4). In the two structures, the latter connection along with O4-H4A⋯O3 hydrogen bond that connected protonated drug cation and water molecule, on the other hand, was involved in the other amide four-membered C(4) motif (Figure 5c). Overall, each drug cation in both structures represented a tri-connected node within this hydrogen bond chain structure and was linked to one chloride anion and one water molecule. The counterion was a two-connected node and functions as a bridge between two drug cations. The water molecule connected to the drug cation was a one-connected node. Therefore, the topology of the trinodal 1,2,3-connected 1D net of (II) could be described as C1_1_.3_2_.4_3_ (Figure 5b), and that of (V) as C1_1_.2_2_.3_3_ (Figure 5d). This tortuous chain propagated parallel to [010].

**Figure 6:**
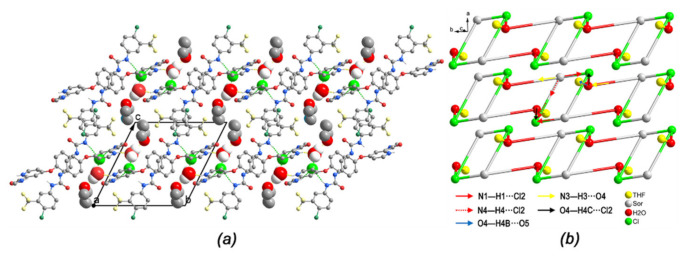
(**a**) Part of the crystal packing of (III), hydrogen bonds are shown as green and yellow dashed lines, H atoms not involved in hydrogen bond have been omitted for clarity; (**b**) 1,3,3,3-Connected tetranodal topological net representing the hydrogen bond chain structure of (III).

In (III), the 1D chain structure was formed by two types of hydrogen bond motifs. The N1 atom on the biuryl group and the N4 atom on the amide group were hydrogen bond donors, and two adjacent cations were bridged by two chloride anions which act as hydrogen bond acceptors. Two N1-H1···Cl2 hydrogen bonds and two N4-H4···Cl2 hydrogen bonds (Table 5) resulted in the $R_{4}^{2}\left(32\right)$ ring motif, forming Sor.HCl-dimer, as was similar to the case of form (I) structure. Additionally, two neighbour dimers were linked by two water molecules through N3-H3···O4 and O4-H4···Cl2, generating a new $R_{6}^{4}\left(18\right)$ pattern. Then the water molecule further donated one hydrogen bond to THF, resulting in the intermolecular interaction O4-H4···O5 (Figure 6a). Altogether, each Sor.H^+^ cation was hydrogen-bonded to three others (two chloride anions and one water); one chloride anion was connected to two drug cations and one water molecule. In the resulting tetranodal 1,3,3,3-connected net, one water molecule served as an additional bridge for Sor.HCl-dimer and one THF molecule via three hydrogen bonds. The topology of the 1,3,3,3-connected net could be described as C1_1_.3_3_.3_3_.3_3_. This chain lied parallel to the [010] (Figure 6b).

**Figure 7:**
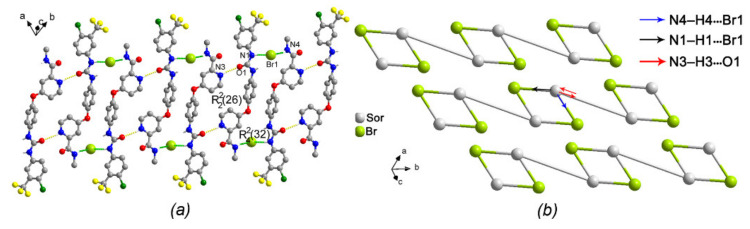
(**a**) Part of the crystal packing of (IV), hydrogen bonds are shown as green and yellow dashed lines, H atoms not involved in hydrogen bond have been omitted for clarity; (**b**) 2,3-Connected dinodal topological net representing the hydrogen bond chain structure of (IV).

Moreover, (IV) and sorafenib hydrochloride were also isomorphous, which were refined in the same space group P$\bar{1}$ (triclinic), with comparable cell dimensions [14]. The hydrogen bonds in the crystal structure of (IV) had been investigated. Similar to sorafenib hydrochloride, two protonated drug cations and two bromide anions created a centrosymmetric dimer-like (Sor.HBr-dimer) ring motif by means of N1–H1···Br1 and N4–H4···Br1 hydrogen bonds (Figure 7a). Two Sor.HBr-dimer rings were then connected directly through O1 atom (N3–H3···O1^i^, forming an $R_{2}^{2}\left(26\right)$ pattern see Table 6 for symmetry code). Therefore, each drug cation of (IV) represented a di-connected node, with one being drug-drug connection through one hydrogen bond and the other being drug cation and bromide anion connection through two hydrogen bonds involving two bromide anions (Figure 7b). The topology of the dinodal 2,3-connected 1D net of (IV) thus could be described as C2_2_.3_3_, and this zigzag chain propagates parallel to [010].

### 2.5. Energy Framework

**Figure 8:**
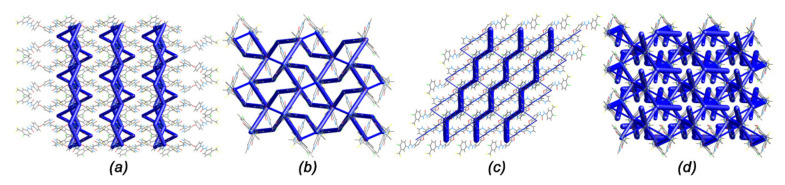
Energy frameworks for the crystal structures of (**a**) (I), (**b**) (II), (**c**) (IV) and (**d**) (V) view down [001] direction. The energy scale factor is 150, and interaction energies with magnitudes smaller than 10 kJ·mol^−1^ have been omitted.

To predict the mechanical property of (I), (II), (IV) and (V), their energy frameworks were presented (Figure 8). (I) showed the zigzag tubes intersecting along the [010] direction. Its energy framework diagram suggested that the slabs were parallel to the [100] direction, comprising molecules linked by very weak interactions. (IV) also showed the zigzag chains stacking along [010] direction, thus (I) and (IV) might bend on the {100} face. In contrast, (II) displayed a complex energy framework with chair-fashion network packing accompanied by slightly weaker crosslinkers that were formed between dimers of Sor.HCl by π···π interactions (the distance between C9-C14 plane and centroid of C1-C6 ring (symmetry code: 1-*x*,1-*y*,1-*z*) is 3.899 Å). (V) presented a similar but more complex energy framework than (II), suggesting that (II) and (V) had an isotropic mechanical behaviour. These results showed that (I) and (IV) might have a better tabletability than (II) and (V).

### 2.6. Solubilities and Dissolution Rates of Sor and Its Salts

**Figure 9:**
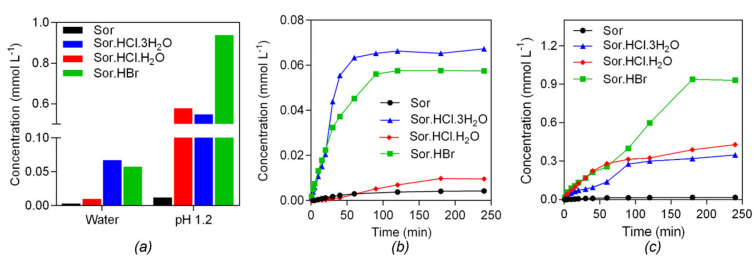
(**a**) Solubility and dissolution rate of (I), (II), (IV) and Sor in (**b**) water and (**c**) pH 1.2 solution.

We tested the solubility and dissolution rate of (I), (II), (IV) and Sor in water and simulated gastric juice (pH 1.2 solution). The solubility of (I), (II) and (IV) was significantly improved in both media (Figure 9a). In water, (I) achieved the highest solubility; and its dissolution rate in this medium was higher than that of (II) and (IV). In contrast, the solubility of (IV) was significantly increased in gastric juice compared with that of (I) and (II), and (IV) dissolved faster than (I) and (II) after 90 *min* of dissolution (Figure 9b,c). In both solutions, the burst release were found to be in the initial stage of the dissolution curves of (I), (II) and (IV).

## 3. Materials and Methods

### 3.1. Synthesis and Crystallization

Crystals of this series of sorafenib salts (I)–(V) were prepared in the mixed solvent. In brief, sorafenib (0.1 g) was added into a 50 mL beaker, and then a mixed solvent of HBr or HCl (with an excess amount of acid) and THF solvent was added. The reaction mixture was stirred at 50 °C until the solids dissolved completely. The resulting solution was then cooled to 0–15 °C for 3–14 days, and the crystals of salts were obtained. The optical micrographs of five forms were then given using Leica DMLB&DMIL at a magnification of 40×.

### 3.2. Powder X-ray Diffraction (PXRD)

The diffraction patterns were acquired on a Rigaku D/Max-2550PC diffractometer. A rotating-anode Cu-target X-ray (λ = 1.5406 Å) was used and operated at 40 kV and 250 mA. The samples were measured from 5.0 to 40.0° with a scanning speed of 5°/min. MERCURY 3.8 was used to attain the calculated diffraction patterns of salts from their single crystal X-ray diffraction (SC-XRD) data.

### 3.3. Single-Crystal X-ray Diffraction (SC-XRD)

SC-XRD data of five sorafenib salts were carried out by a Bruker APEX-II CCD (Mo Kα radiation, λ = 0.71073 Å) [18]. Integration and scaling of intensity data were accomplished using the SAINT program [19]. The structures were solved by the direct method, refined and graphed molecularly using SHELXL-97 [20,21]. Then Mercury 3.8 was utilized to analyse the supramolecular hydrogen bond and molecular conformation, and the graphs were presented using DIAMOND [22]. The topology of the hydrogen bond structures was determined and classified with the programs ADS and IsoTest of the TOPOS package [23] in the manner described by Baburin & Blatov [24]. Predicted morphology was calculated using Materials Studio 7.0 [25]. To calculate the intermolecular interaction energy, B3LYP-D2/6-31G(d,p) model based on dispersion-corrected density functional theory was employed using CrystalExplorer 17 [26]. The “energy framework” was created based on the total intermolecular interaction energy, which included electrostatic, polarization, dispersion, and exchange-repulsion components with scale factors of 1.057, 0.740, 0.871 and 0.618, respectively [11].

### 3.4. Solubility and Dissolution Measurement

The solubility and dissolution rate studies of Sor, Sor.HBr, Sor.HCl.H_2_O and Sor.HCl.3H_2_O in water and gastric juice pH 1.2 (in the presence of 0.2% sodium lauryl sulphate-SLS) at 37 ^o^C were conducted by Thermo Scientific Evolution 300 UV–Vis spectrometer (Thermo Scientific Evolution 300, Thermo Scientific, Waltham, MA, USA). For equilibrium solubility, excess quantities of drugs were dispersed in 5 mL of aqueous solution in screw-capped vials and stirred at 100 rpm and 37 °C for 24 h to obtain saturated solutions. Saturated solutions were then filtered through Whatman’s 0.45 μm syringe filter. For dissolution rate measurement, excess quantities of samples were poured into 250 mL of solvent which was preheated to 37 °C and stirred at 150 rpm. In experiments, 2 mL of dissolved sample was withdrawn at specific time intervals for 240 min and replaced with an equal volume of the fresh medium to maintain a constant total volume. All solutions measured the absorbance at their λ_max_.

## 4. Conclusions

In conclusion, we note that sorafenib hydrochloride exists in two hydrate forms and one solvate form which were obtained concomitantly in a mixture of THF and water. We successfully separated them by changing the condition of preparation. Sorafenib hydrobromide and its hydrate form also crystallized concomitantly in THF: water, however, the amount of hydrate form was very little. The theoretical morphologies were predicted based on the BFDH model and found useful to identify these forms. The hydrogen bond systems showed that the role of water in stabilizing the crystal structures was different, and the topologies indicated that these systems were not similar. The energy framework suggested that Sor.HCl.3H_2_O and Sor.HBr was more tabletable than Sor.HCl.H_2_O and Sor.HBr.H_2_O. Moreover, Sor.HCl.3H_2_O, Sor.HCl.H_2_O and Sor.HBr had a higher solubility and dissolution rate than Sor in water and gastric juice (pH 1.2).

## Figures and Tables

**Table 1:** Crystal data.

	(I)	(II)	(III)	(IV)	(V)
Chemical formula	Cl·C_21_H_17_ClF_3_N_4_O_3_·3(H_2_O)	(Cl)·(C_21_H_17_ClF_3_N_4_O_3_)·(H_2_O)	Cl·C_21_H_17_ClF_3_N_4_O_3_·H_2_O·C_4_H_8_O	Br·C_21_H_17_ClF_3_N_4_O_3_	Br·C_21_H_17_ClF_3_N_4_O_3_·H_2_O
*M*_r_	555.33	2077.20	591.40	545.74	563.76
Crystal system, space group	Monoclinic, *P*2_1_/*c*	Monoclinic, *P*2_1_/n	Triclinic, *P*$\bar{1}$	Triclinic, $P\bar{1}$	Monoclinic, *P*2_1_/n
*a*, *b*, *c* (Å)	13.9736 (5), 12.7166 (5), 14.6215 (5)	11.3049 (3), 8.5008 (2), 23.1013 (6)	9.1309 (3), 13.0397 (4), 13.2554 (5)	9.545 (3), 10.840 (3), 12.353 (4)	11.3349 (15), 8.7524 (10), 23.048 (3)
α, β, γ (°)	90, 103.294 (1), 90	90, 93.826 (1), 90	63.472 (1), 89.255 (1), 88.985 (1)	77.633 (13), 87.153 (16), 64.131 (12)	90, 93.167 (5), 90
*V* (Å^3^)	2528.57 (16)	2215.10 (10)	1411.84 (8)	1121.9 (6)	2283.1 (5)
*Z*	4	1	2	2	4

**Table 2:** Hydrogen-bond geometry (Å, º) for (I).

*D*—H···*A*	*D*—H	H⋯*A*	*D*⋯*A*	*D*—H⋯*A*
N1—H1···Cl2	0.86	2.42	3.2473 (14)	161
N2—H2···Cl2	0.86	2.44	3.2527 (14)	158
N4—H4···Cl2 ^i^	0.86	2.41	3.2122 (16)	156
O4—H4*A*···Cl2 ^ii^	0.85	2.38	3.2049 (17)	163
O4—H4*B*···O3 ^iii^	0.85	1.96	2.796 (2)	169
O5—H5*A*···O4	0.85	1.96	2.764 (3)	158
O5—H5*B*···O6	0.85	1.89	2.737 (3)	172
O6—H6*A*···O1	0.85	2.02	2.869 (2)	175
N3—H3···O5 ^iv^	0.92 (2)	1.77 (3)	2.631 (2)	154 (2)
O6—H6*B*···Cl2 ^v^	0.89 (4)	2.34 (4)	3.225 (2)	172 (3)

Symmetry codes: ^i^ −*x* + 1, −*y* + 1, −*z* + 1; ^ii^ −*x* + 1, *y*−1/2, −*z* + 3/2; ^iii^ −*x* + 2, −*y* + 1, −*z*+1; ^iv^ −*x* + 2, *y* + 1/2, −*z* + 3/2; ^v^ −*x* + 1, −*y* + 1, −*z* + 2.

**Table 3:** Hydrogen-bond geometry (Å, º) for (II).

*D*—H⋯*A*	*D*—H	H⋯*A*	*D*⋯*A*	*D*—H⋯*A*
N1—H1⋯Cl2	0.88	2.35	3.1934 (16)	161
N2—H2⋯Cl2	0.88	2.47	3.2872 (15)	155
N4—H4⋯Cl2 ^i^	0.88	2.45	3.2569 (16)	153
O4—H4*B*⋯O3	0.97 (4)	2.12 (7)	2.774 (3)	124 (6)

Symmetry code: ^i^ −*x* + 1/2, *y* + 1/2, −*z* + 3/2.

**Table 4:** Hydrogen-bond geometry (Å, º) for (V).

*D*—H⋯*A*	*D*—H	H⋯*A*	*D*⋯*A*	*D*—H⋯*A*
N4—H4⋯Br1 ^i^	0.88	2.59	3.3853 (18)	151
N1—H1⋯Br1	0.88	2.49	3.331 (2)	161
O4—H4*B*⋯O3 ^ii^	0.94 (2)	2.23 (5)	2.868 (3)	125 (4)

Symmetry codes: ^i^ −*x* + 3/2, *y* − 1/2, −*z* + 1/2; ^ii^ −*x* + 1, −*y* + 2, −*z*.

**Table 5:** Hydrogen-bond geometry (Å, º) for (III).

*D*—H⋯*A*	*D*—H	H⋯*A*	*D*⋯*A*	*D*—H⋯*A*
O4—H4*B*⋯O5	0.85	1.83	2.676 (4)	171
O4—H4*C*⋯Cl2	0.85	2.32	3.118 (2)	156
N4—H4⋯Cl2 ^ii^	0.86	2.42	3.206 (2)	152
N1—H1⋯Cl2 ^i^	0.86	2.38	3.224 (2)	168
N3—H3⋯O4	0.89 (3)	1.79 (3)	2.641 (3)	159 (2)

Symmetry codes: ^i^ *x*, *y* − 1, *z*; ^ii^ −*x*, −*y* + 2, −*z* + 1.

**Table 6:** Hydrogen-bond geometry (Å, °) for (IV).

*D*—H⋯*A*	*D*—H	H⋯*A*	*D*⋯*A*	*D*—H⋯*A*
N1—H1⋯Br1 ^i^	0.88	2.48	3.324 (2)	160
N3—H3⋯O1 ^ii^	0.88	2.01	2.860 (2)	162
N4—H4*A*⋯Br1	0.88	2.48	3.325 (3)	161

Symmetry codes: ^i^ −*x*, −*y* + 1, −*z* + 1; ^ii^ −*x* + 1, −*y*, −*z* + 1.

## Data Availability

CCDC Number: 2077873-2077877 with doi: 10.5517/ccdc.csd.cc27r65d, 10.5517/ccdc.csd.cc27r66f, 10.5517/ccdc.csd.cc27r67g, 10.5517/ccdc.csd.cc27r68h, 10.5517/ccdc.csd.cc27r69j.

## Supplementary Materials

The following are available online at https://www.mdpi.com/article/10.3390/ijms22136682/s1.
