# Supplementary material for: Hydrogen Bonds, Topologies, Energy Frameworks and Solubilities of Five Sorafenib Salts
Source: Int J Mol Sci. 2021 Jun 22;22(13):6682. doi: 10.3390/ijms22136682 (PMC8268146; doi:10.3390/ijms22136682)
Supplement: Supplementary file 1 [file ijms-22-06682-s001.zip › ijms-1243486-supplementary.pdf]

Supporting information

Hydrogen bonds, topologies, energy frameworks and solubilities of five sorafenib salts

Authors

Chiuyen Phan[a]*, Jie Shen[b], Kaxi Yu[b], Jiyong Liu[b] and Guping Tang[b]*

[a]Faculty of Chemical Technology - Environment, University of Technology and Education, The University of Danang, Danang, 550000, Vietnam

[b]Department of Chemistry, Zhejiang University, Hangzhou, Zhejiang, 310028, People's Republic of China

Correspondence email: pcuyen@ute.udn.vn; tangguping@zju.edu.cn

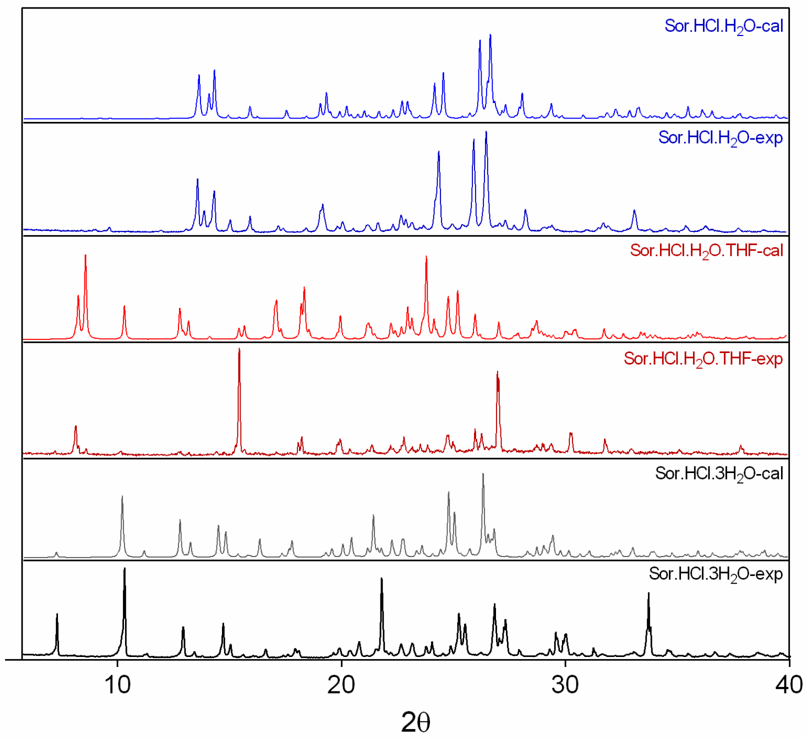

Figure S1 Overlay of experimental and simulated PRXD patterns of (I) – (III)
